# Functionality of Redox-Active Cysteines Is Required for Restriction of Retroviral Replication by SAMHD1

**DOI:** 10.1016/j.celrep.2018.06.090

**Published:** 2018-07-24

**Authors:** Zhonghua Wang, Akash Bhattacharya, Tommy White, Cindy Buffone, Aine McCabe, Laura A. Nguyen, Caitlin N. Shepard, Sammy Pardo, Baek Kim, Susan T. Weintraub, Borries Demeler, Felipe Diaz-Griffero, Dmitri N. Ivanov

**Affiliations:** 1 Department of Biochemistry & Structural Biology, University of Texas Health Science Center, San Antonio, TX 78229, USA; 2 Department of Microbiology and Immunology, Albert Einstein College of Medicine, Bronx, NY 10461, USA; 3 Center for Drug Discovery, Department of Pediatrics, Emory School of Medicine, Atlanta, GA 30322, USA; 4 School of Pharmacy, Kyunghee University, Seoul, South Korea

## Abstract

SAMHD1 is a dNTP triphosphohydrolase (dNTPase) that impairs retroviral replication in a subset of noncycling immune cells. Here we show that SAMHD1 is a redox-sensitive enzyme and identify three redox-active cysteines within the protein: C341, C350, and C522. The three cysteines reside near one another and the allosteric nucleotide binding site. Mutations C341S and C522S abolish the ability of SAMHD1 to restrict HIV replication, whereas the C350S mutant remains restriction competent. The C522S mutation makes the protein resistant to inhibition by hydrogen peroxide but has no effect on the tetramerization-dependent dNTPase activity of SAMHD1 *in vitro* or on the ability of SAMHD1 to deplete cellular dNTPs. Our results reveal that enzymatic activation of SAMHD1 via nucleotide-dependent tetramerization is not sufficient for the establishment of the antiviral state and that retroviral restriction depends on the ability of the protein to undergo redox transformations.

## In Brief

SAMHD1 impedes infection of noncycling immune cells by HIV-1. Wang et al. identify three redox-active cysteines in SAMHD1 and demonstrate that the antiretroviral activity of SAMHD1 depends on the ability of the protein to undergo redox transformations.

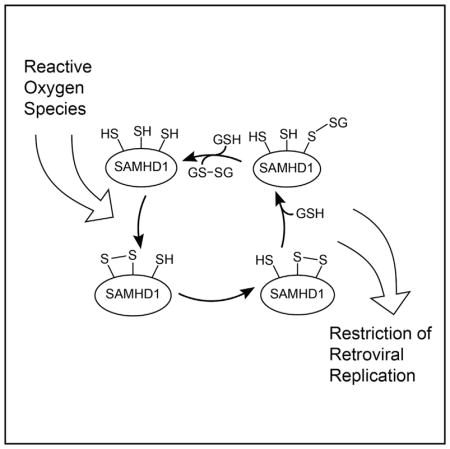

## INTRODUCTION

Discoveries that SAMHD1 mutations cause Aicardi-Goutières syndrome (AGS) (Rice et al., 2009) and that SAMHD1 restricts HIV-1 replication in non-cycling immune cells (Baldauf et al., 2012; Berger et al., 2011; Descours et al., 2012; Hrecka et al., 2011; Laguette et al., 2011) established the role of SAMHD1 as an innate immunity factor involved in interferon signaling and retroviral restriction.

SAMHD1 is a member of the HD domain family of enzymes, and the protein displays nucleotide-dependent dinucleotide triphosphate triphophohydrolase (dNTPase) activity previously described for other members of this protein family (Lahouassa et al., 2012; Powell et al., 2011; Goldstone et al., 2011; Beauchamp and Richardson, 1988; Kornberg et al., 1958; Seto et al., 1988). The dNTPase activity of SAMHD1 is thought to be central for its antiretroviral function, because SAMHD1 blocks retroviral replication before completion of reverse transcription (Fujita et al., 2008; Goujon et al., 2007; Laguette et al., 2011) and the SAMHD1-mediated decrease in the dinucleotide triphosphate (dNTP) levels in myeloid cells correlates with the inability of lentiviruses to undergo reverse transcription (Kim et al., 2012; Lahouassa et al., 2012). However, the details of the retroviral restriction mechanism remain elusive, and it is debated whether the SAMHD1-catalyzed depletion of cellular dNTPs is sufficient for retroviral restriction or whether some additional and distinct SAMHD1 functionality may be involved (Cribier et al., 2013; Welbourn et al., 2013; White et al., 2013b; Antonucci et al., 2016; Ryoo et al., 2014, 2016; Seamon et al., 2015; Welbourn and Strebel, 2016; Bhattacharya et al., 2016; Brandariz-Nuñez et al., 2013).

Outstanding questions concern the exact relationship between enzymatic properties of SAMHD1 and its function as an immune factor. For example, the dNTPase activity of SAMHD1 depends on binding of nucleotides at two distinct allosteric sites and on transient tetramerization of the protein (Ji et al., 2013, 2014; Koharudin et al., 2014; Yan et al., 2013; Zhu et al., 2013). Surprisingly, several tetramerization-defective mutants of SAMHD1 that display a striking dNTPase defect *in vitro* are nevertheless intact in their ability to deplete dNTPs in immune cells and to restrict retroviral replication (Bhattacharya et al., 2016; Brandariz-Nuñez et al., 2013). Another poorly understood aspect of SAMHD1 function involves protein phosphorylation on threonine 592 (T592). T592 is phosphorylated in cycling cells, and the phosphate group needs to be removed to enable the restriction activity of SAMHD1 (Cribier et al., 2013; Welbourn et al., 2013; Welbourn and Strebel, 2016; White et al., 2013b). Phosphomimetic mutations of T592 abolish restriction but do not affect the dNTPase activity of the protein *in vitro* or its ability to deplete dNTPs in cells (Bhattacharya et al., 2016; Welbourn et al., 2013; White et al., 2013b), raising additional questions about the relationship between dNTPase activity and restriction. Finally, SAMHD1 is known to interact with nucleic acids (Beloglazova et al., 2013; Goncalves et al., 2012; Seamon et al., 2015; Tungler et al., 2013) and to contribute to DNA double-strand break repair (Daddacha et al., 2017) in a dNTPase-independent manner, but it remains unknown whether these additional functionalities contribute to its antiretroviral function.

This study emerged from our efforts to identify additional factors that modulate enzymatic properties of SAMHD1. Here we report that enzymatic activity of SAMHD1 depends on its oxidation state and establish that the ability of SAMHD1 to undergo redox transformations is required for the antiretroviral activity of the protein.

## RESULTS

### SAMHD1 Is a Redox-Sensitive Enzyme that Forms Covalent Conjugates with Glutathione In Vitro and in Immune Cells

A screen for factors affecting dNTPase activity of SAMHD1 revealed that the enzyme is sensitive to the choice of the reducing agent (Figure S1). The effect was strongest for full-length SAMHD1 immunopurified from mammalian cells. We observed that addition of 5 mM DTT or 5 mM reduced glutathione to the reaction buffer dramatically increased dNTPase activity of SAMHD1 (Figure S1A), whereas brief incubation with hydrogen peroxide inhibited the enzyme (Figure S1B).

**Figure 1:**
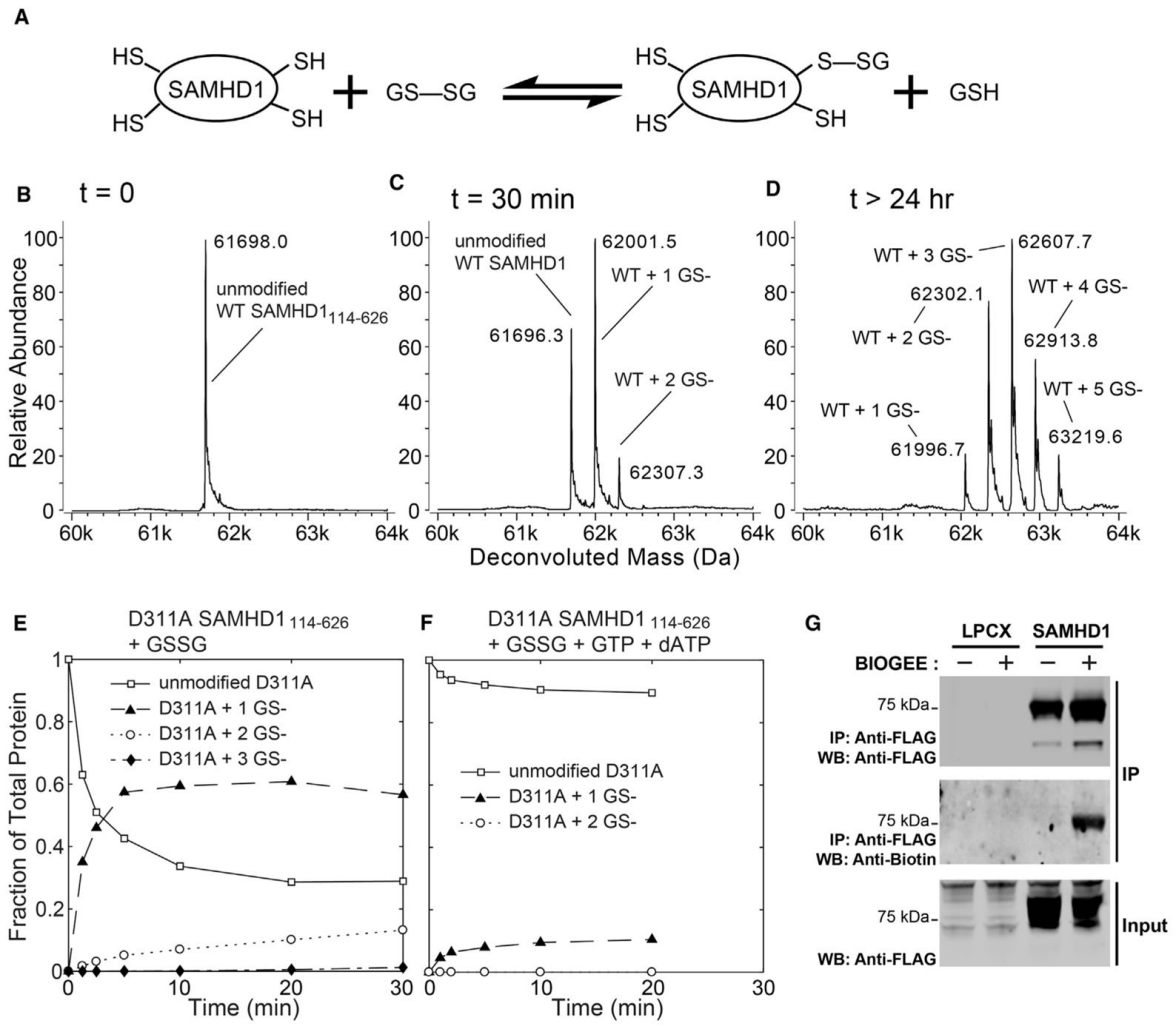
SAMHD1 S-Glutathionylation *In Vitro* and in Cultured U937 Cells (A) Disulfide exchange reactions with oxidized glutathione can be used to identify redox-active cysteines in proteins. (B–D) Analysis of SAMHD1 S-glutathionylation by intact-mass ESI-TOF mass spectrometry. Deconvoluted mass spectra of the purified HD domain construct of SAMHD1_114–626_ before (B) and after (C) a 30-min incubation with 5 mM oxidized glutathione reveal formation of covalent glutathione conjugates. (D) Overnight incubation with oxidized glutathione results in modification of the protein with up to five glutathione molecules per SAMHD1 monomer. (E and F) Time course experiments reveal that SAMHD1 glutathionylation depends on its oligomerization state. Rapid monoglutathionylation of the catalytically inactive mutant D311A (E) is abolished upon addition of GTP and dATP nucleotides, which promote SAMHD1 tetramerization (F). (G) Glutathionylation of immunopurified full-length SAMHD1-FLAG can be detected by western blot analysis after incubation of SAMHD1-expressing, PMA-treated human U937 cells with biotinylated glutathione (BIOGEE). See also Figures S1 and S3.

Redox sensitivity of protein activity most frequently involves surface-exposed cysteine residues, which can be identified by their propensity to participate in thiol-disulfide exchange reactions (Shelton et al., 2005). We first investigated the ability of the protein to undergo S-glutathionylation upon incubation with oxidized L-glutathione (GSSG) *in vitro* (Figure 1A). The HD domain construct of SAMHD1 (SAMHD1_114–626_) was incubated with oxidized glutathione and analyzed on an electrospray ionization time-of-flight mass spectrometry (ESI-TOF) mass spectrometer. We observed that 30-min incubation with oxidized glutathione resulted in more than 50% of total protein forming a monoglutathionylated adduct (Figures 1B and 1C), whereas overnight incubation resulted in multi-glutathionylation, with up to five glutathione molecules conjugated to the protein (Figure 1D). Five of ten cysteines in the HD domain are solvent accessible, and the predominance of the monoglutathionylated species after a 30-min incubation suggested that one of the surface exposed cysteines is significantly more reactive than the others.

We then investigated whether the catalytic metals or nucleotide-dependent tetramerization contribute to redox reactivity of SAMHD1_114–626_. The D311A mutation that disrupts metal coordination in the active site had no effect on glutathione conjugation. Monoglutathionylation of D311A is faster than diglutathionylation, supporting the existence of a single reactive cysteine residue within the protein (Figure 1E). Rapid monoglutathionylation of SAMHD1 is abolished in the presence of guanosine triphosphate (GTP) and deoxyadenosine triphosphate (dATP)—allosteric activators that promote SAMHD1 tetramerization—revealing that redox reactivity of SAMHD1 is inhibited by nucleotide-dependent tetramerization (Figure 1F).

We then evaluated whether SAMHD1 glutathionylation can be detected in cells. Incubation of phorbol-12-myristate-3-acetate (PMA)-treated human U937 cells stably expressing full-length SAMHD1-FLAG with biotinylated glutathione revealed that SAMHD1 glutathionylation occurs in cultured U937 cells (Figure 1G). However, we were not able to reliably detect SAMHD1 glutathionylation in primary macrophages using this method. In primary cells, the uptake of biotinylated glutathione from the media may be inefficient, or the S-glutathionylated intermediate of SAMHD1 may be short lived.

### C341, C350, and C522 Are Identified as Redox-Active Cysteines by Mass Spectrometry

**Figure 2:**
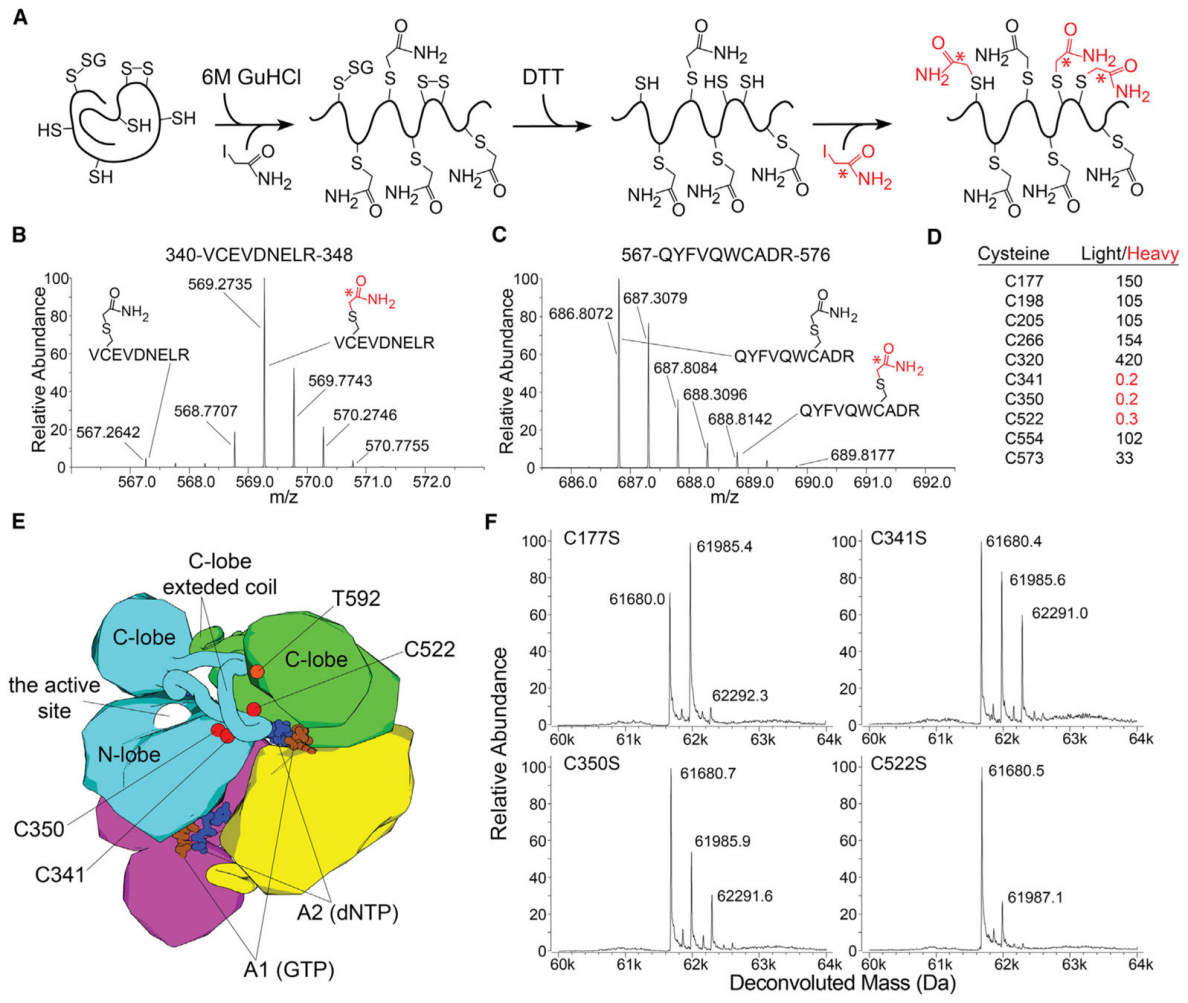
Identification of the Redox-Active Cysteines by Mass Spectrometry (A) Experimental procedure for quantifying cysteine oxidation in the protein treated with oxidized glutathione. The quantitative proteomics analysis relies on the differential labeling of the protein with the light and heavy forms of iodoacetamide. (B–D) All cysteine-containing peptides were observed to fall into two distinct classes: peptides predominantly labeled with the heavy iodoacetamide (B) and peptides predominantly labeled with the light form (C). (D) Typical light-to-heavy abundance ratios measured for peptides containing each of the 10 cysteines in SAMHD1. (E) Cartoon depiction of the SAMHD1 tetramer fully loaded with allosteric ligands as observed by X-ray crystallography (PDB: 4BZC). The three redox-active cysteines reside near the tetramerization interface, the allosteric binding sites, and the T592 residue. The proximity of C341 and C350 to each other favors formation of the intramolecular disulfide bond. (F) Effect of cysteine mutations on SAMHD1 glutathionylation as evaluated by intact-mass analysis (ESI-TOF). See also Figures S2 and S3.

We used quantitative mass spectrometry (MS) to measure cysteine oxidation after 30 min of incubation with oxidized glutathione (Figure 2A; Supplemental Experimental Procedures). Clear differences in isotopic labeling patterns revealed that three cysteines—C341, C350, and C522—become oxidized (Figures 2B–2D), which was surprising because the protein is predominantly monoglutathionylated after the glutathione treatment.

Site-directed mutagenesis was used to evaluate the contribution of the three redox-active cysteines to SAMHD1 glutathionylation (Figure 2F). Mutation of a non-redox-active surface-exposed cysteine, C177S, was used as a control and behaved similarly to wild-type (WT) protein. In contrast, C522S mutation resulted in a significant reduction of protein glutathionylation. The C341S and C350S mutations had a different effect: they significantly increased the amount of diglutathionylated species of SAMHD1 after 30 min of incubation. The distinct phenotypes of C522S, C341S, and C350S mutants were confirmed by measurement of the glutathionylation rates, which revealed a pronounced reduction in the rate of monoglutathionylation of C522S and an increase in the diglutathionylation rate in C341S and C350S (Figure S2). In agreement with the *in vitro* studies, we observed reduced reactivity of C522S SAMHD1 with biotinylated glutathione in cultured U937 cells (Figure S3). Collectively, the data suggest that treatment of SAMHD1 with oxidized glutathione results in rapid glutathionylation of C522 and in formation of the C341-C350 disulfide. Formation of the C341-C350 disulfide had been previously observed in several crystal structures of SAMHD1 (PDB: 3U1N, 4MZ7, 4Q7H, 4RXO, 4QG1, and 4QG2) (Goldstone et al., 2011; Koharudin et al., 2014; Zhu et al., 2013, 2015).

### C341S and C350S Mutations Diminish SAMHD1 Tetramerization and dNTPase Activity, whereas C522S Is Fully Functional and Makes the Protein Resistant to Inhibition by Hydrogen Peroxide

**Figure 3:**
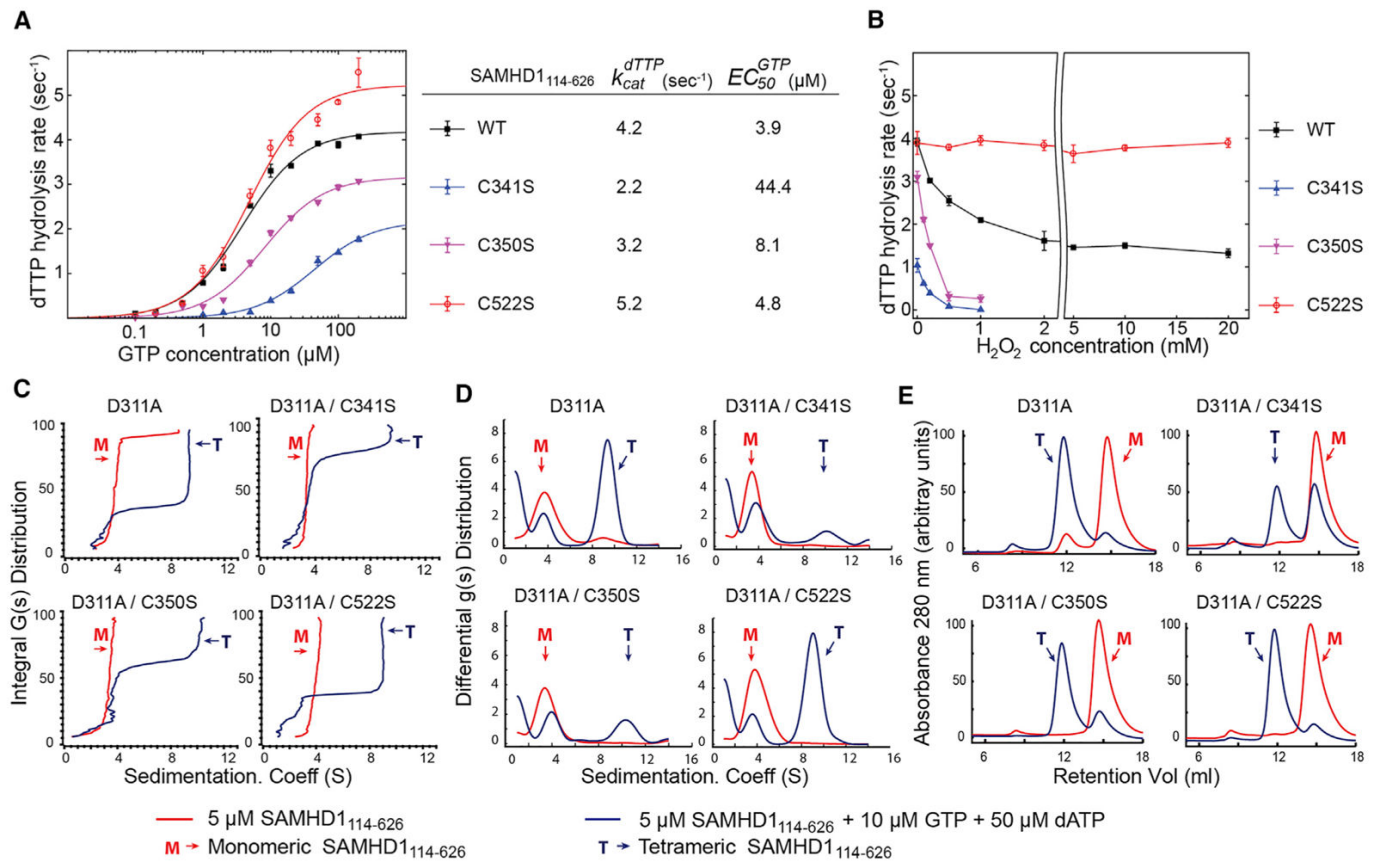
Effects of Cysteine Mutations on the dNTPase Activity and Tetramerization of SAMHD1_114–626_ (A) dNTPase activity of SAMHD1 mutants evaluated as a function of GTP concentration using an NMR-based dNTPase assay. *k_cat_* and EC_50_*^GTP^* parameters were determined by non-linear fitting of the data to the simple EC_50_ equation. (B) H_2_O_2_ sensitivity of SAMHD1 cysteine mutants. After 3 min of incubation with increasing concentrations of H_2_O_2_, the dTTP hydrolysis rate was measured in the presence of 50 μM GTP. (C and D) Analytical ultracentrifugation studies of the catalytically inactive SAMHD1 variants. Sedimentation velocity runs were performed with protein alone (red traces) and in the presence of nucleotides (blue traces). Sedimentation velocity analysis showing diffusion-corrected van Holde-Weischet integral G(s) distributions (C) and differential g(s) distributions (D) obtained from the parametrically constrained spectrum analysis. (E) Size-exclusion chromatography analysis of SAMHD1 tetramerization is in agreement with the AUC results. n = 2, where n represents the number of independent measurements of the rate. Error bars denote SD.

We then evaluated the impact of cysteine mutations on the dNTPase activity and on the ability of the protein to tetramerize in the presence of allosteric ligands. The dNTPase activity was investigated by measuring dNTP hydrolysis rates as a function of GTP concentration. These curves are informative of both the tetramerization propensity of the protein and the *k_cat_* of the catalytically active tetramer (Bhattacharya et al., 2016). The experiments revealed that catalytic and tetramerization properties of the C522S mutant were indistinguishable from those of the WT protein. In contrast, the C341S and C350S mutations displayed approximately 50% and 25% reductions in the maximal catalytic rate of the protein, respectively. The apparent half-maximal effective concentration (EC_50_) values for GTP-mediated activation for C341S and C350S were also higher than in the WT protein (Figure 3A).

We also measured the effect of hydrogen peroxide treatment on the dNTPase activity of the cysteine mutants (Figure 3B). These experiments revealed that the dNTPase activity of WT, C341S, and C350S proteins displayed significant reduction of their catalytic activity with increasing hydrogen peroxide concentrations. In contrast, C522S was resistant to inhibition by hydrogen peroxide and displayed almost full activity even at the highest hydrogen peroxide concentrations tested.

Finally, the effect of cysteine mutations on nucleotide-dependent tetramerization of SAMHD1 was evaluated using sedimentation velocity analytical ultracentrifugation and size-exclusion chromatography (Figures 3C–3E). These experiments were performed on the catalytically inactive SAMHD1_114–626_ D311A variant. The D311A mutation does not affect the tetramerization properties of SAMHD1_114–626_ (Bhattacharya et al., 2016) and was used to prevent dNTP depletion by the catalytically active SAMHD1 tetramer. In agreement with the dNTPase activity assays, these experiments revealed that the D311A/C522S mutant was indistinguishable from the D311A protein in its tetramerization ability, in contrast to D311A/C341S and D311A/ C350S mutants, which displayed a reduced tetramerization capacity.

### C522S and C341S, but Not C350S, Abolish SAMHD1-Mediated Retroviral Restriction, whereas Depletion of dNTP Pools, T592 Dephosphorylation, and In-Cell Oligomerization of SAMHD1 Are Not Affected by Cysteine Mutations

**Figure 4:**
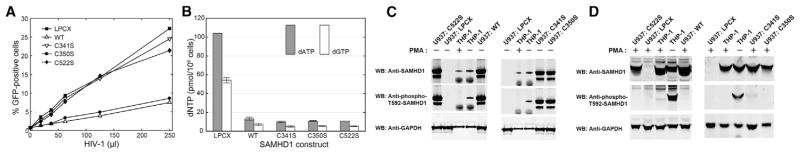
Effects of Cysteine Mutations on the Restriction Activity, dNTP Depletion, and T592 Dephosphorylation (A) PMA-treated U937 cells stably expressing the indicated SAMHD1 variants were challenged with increasing amounts of HIV-1-GFP. Infection was determined by measuring the percentage of GFP-positive cells using flow cytometry. n = 3, where n denotes the number of independent viral challenges with all mutants, but only one representative set of challenges is shown. The same mutant-dependent trends were observed in all three replicate experiments. (B) Total cellular levels of dATP and deoxyguanosine triphosphate (dGTP) were measured in PMA-treated U937 cells stably expressing SAMHD1 variants using a primer extension assay, as described in Experimental Procedures. n = 3, where n represents the number of independent dNTP concentration measurements using the primer extension assay. Error bars denote SD. (C and D) Cysteine mutations do not affect dephosphorylation of T592 following PMA treatment of U937 cells stably expressing WT and mutant SAMHD1 variants. (C) T592 is phosphorylated in U937 cells expressing SAMHD1 and not treated with PMA in all SAMHD1 variants tested. SAMHD1 expression levels are low in U937 cells before PMA treatment, so the phosphorylation status was evaluated on immunoprecipitated SAMHD1 (see Experimental Procedures). (D) Following PMA treatment, dephosphorylation of T592 is evident in the WB analysis of the crude cell extracts. Cysteine mutations have no effect on T592 dephosphorylation. Dephosphorylation of the endogenous SAMHD1 in PMA-treated and untreated THP-1 cells is shown as control in all gels. See also Figure S4.

Retroviral restriction by cysteine mutants was evaluated in PMA-treated U937 cells stably expressing the different SAMHD1 variants that were challenged with increasing amounts of HIV-1 virus expressing GFP as a reporter of infection (Figure 4A). These studies revealed that C522S and C341S mutations abolish retro-viral restriction, whereas the C350S is indistinguishable from the WT protein. The restriction defect of the C522S mutation is particularly intriguing, because it has no detectable detrimental effect on SAMHD1 tetramerization and dNTPase activity *in vitro*.

The effect of cysteine mutations on the ability of SAMHD1 to deplete dNTPs in PMA-treated U937 cells expressing WT and mutant SAMHD1 constructs was also evaluated as previously described (White et al., 2013a, White et al., 2013b). These experiments revealed that none of the cysteine mutations have a significant effect on the ability of SAMHD1 to deplete dNTPs (Figure 4B).

The apparent lack of correlation between dNTPase activity, dNTP depletion, and HIV restriction observed for the C522S mutant parallels the phenotype of the phosphomimetic mutations T592D and T592E. To investigate whether redox transformations are linked to T592 phosphorylation status in SAMHD1, we measured the phosphorylation levels of T592 in the cysteine mutants by western blotting using anti-phospho-T592 antibodies in untreated and PMA-treated U937 cells (Figures 4C and 4D), as previously described (White et al., 2013b). SAMHD1 expression in U937 cells before PMA treatment is low, so SAMHD1 was preconcentrated by immunoprecipitation before western blot (WB) analysis of its phosphorylation status in cells not treated with PMA (Figure 4C). In PMA-treated cells, SAMHD1 phosphorylation status was tested in crude cell extracts (Figure 4D). We observed that the cysteine mutations did not affected T592 phosphorylation. These experiments suggested that the inability of SAMHD1 C341S and C522S to block HIV-1 infection is not due to a change in the phosphorylation state of SAMHD1.

Finally, we tested the ability of SAMHD1 variants to oligomerize in mammalian cells, as previously described (White et al., 2013b). None of the tested SAMHD1 variants showed a defect in oligomerization when compared to WT SAMHD1 (Figure S4). As control, we used the SAMHD1 variant Y146S/Y154S, which is defective in its ability to oligomerize (Brandariz-Nuñez et al., 2013).

## DISCUSSION

In this study, we establish an essential role of redox transformations in the antiretroviral activity of SAMHD1. Effects of the C522S mutation on SAMHD1 function parallel the puzzling phenotype of the phosphomimetic variants T592E and T592D. The C522S mutation does not affect protein tetramerization or dNTPase activity *in vitro* and does not impair SAMHD1-mediated dNTP depletion in cells; however, it abolishes the ability of the protein to restrict retroviral replication (Cribier et al., 2013; Welbourn et al., 2013; White et al., 2013b; Welbourn and Strebel, 2016). Collectively, properties of the C522S variant, T592 phosphomimetics, and the tetramerization-defective mutant Y146S/Y154S (Bhattacharya et al., 2016; Brandariz-Nuñ ez et al., 2013) demonstrate that SAMHD1 activation via nucleotide-dependent tetramerization is not sufficient for retroviral restriction, and an additional SAMHD1 functionality is needed for the establishment of the SAMHD1-mediated antiretroviral state. One likely possibility is that nucleotide-dependent tetramerization is only one of several ways dNTP hydrolysis by SAMHD1 can be activated and that in restrictive cells, the protein is maintained to be enzymatically active through a distinct mechanism, one that depends on the phosphorylation state of T592 and the redox activity of C522. Better understanding of SAMHD1 allosteric regulation may thus shed light on whether and how the dNTPase activity of SAMHD1 contributes to the various biological functions of the protein.

The presence of multiple redox-active cysteines in SAMHD1, which is unusual for cytoplasmic or nuclear proteins, is suggestive of a regulatory function of these residues. C341 and C350 are ideally positioned to form a disulfide bond linking two adjacent short β strand segments. However, formation of the C341-C350 disulfide is not essential for restriction, because the C350S mutant is fully restriction competent. C522 resides in a distinctive extended coil segment of the SAMHD1 structure, which emanates from the C-terminal lobe of the protein, spans the entire length of the catalytic N-terminal lobe, and brings C522 near the C341-C350 pair and the allosteric nucleotide binding site (Figure 2E). Proximity of the three redox-active residues and loss of restriction in the C522S and C341S variants suggest that a transient C522-C341 disulfide may act as a key intermediate in a disulfide-exchange mechanism reminiscent of redox regulation of Ero1alpha (Appenzeller-Herzog et al., 2008; Ramming et al., 2016).

Several signaling and regulatory pathways have been implicated in controlling SAMHD1 activity (Cribier et al., 2013; Lee et al., 2017; Mlcochova et al., 2017, 2018; Schott et al., 2018). This study describes another tantalizing piece in the complex puzzle of SAMHD1 involvement in antiviral immunity, interferon signaling, suppression of retrotransposons, regulation of cellular dNTP pools, and DNA damage response and repair (Franzolin et al., 2013; Zhao et al., 2013; Daddacha et al., 2017; Coquel et al., 2018). Further investigation of biochemical and immune consequences of SAMHD1 redox transformations may offer insight into the emerging role of reactive oxygen species in immune signaling (Holmström and Finkel, 2014; Nathan and Cunningham-Bussel, 2013) and their potential contribution to the susceptibility of macrophages and resting T cells to HIV infection.

## EXPERIMENTAL PROCEDURES

### Protein Expression and Purification

The WT and mutant variants of full-length SAMHD1 (SAMHD1_1–626_) were expressed and purified from mammalian cells, and the HD domain constructs (SAMHD1_114–626_) were produced using a bacterial expression system as previously described (Bhattacharya et al., 2016; Wang et al., 2016).

### NMR-Based dNTPase Assay

Kinetics of the dNTPase reaction catalyzed by SAMHD1 was investigated using a nuclear magnetic resonance (NMR)-based dNTPase assay (Bhattacharya et al., 2016). All assay mixtures contained 1 mM thymidine triphosphate (dTTP) substrate and 0.2 μM SAMHD1 in the following buffer: 50 mM Tris (pH 7.4), 150 mM NaCl, 5 mM MgCl_2_, and 10% D_2_O. In addition, all samples contained oxidizers or reducing agents as specified in Results. All experiments shown in Figure 4A were carried out in the presence of 5 mM DTT.

### MS

S-glutathionylation of SAMHD1 (Figures 1A–1F and 2F; Figure S2) was investigated by incubating the proteins with 5 mM oxidized glutathione (Acros Organics) for increasing periods of time and analyzing the products by intact-mass MS as described in detail in Supplemental Information.

Quantification of cysteine oxidation (Figures 2A–2D) was carried out by first incubating the protein with 5 mM oxidized glutathione for 30 min. The samples were subsequently treated by unlabeled iodoacetamide, DTT, and isotopically enriched iodoacetamide, as shown in Figure 2A. The protein was then digested with trypsin, and the peptide fragments were analyzed by liquid chromatography-tandem MS (LC-MS/MS) as described in more detail in Supplemental Information. Abundances of light and heavy variants of each carbamidomethylated peptide were quantified and used to determine the degree of oxidation for each cysteine residue.

### Analytical Ultracentrifugation and Size-Exclusion Chromatography

SAMHD1 samples (5 μM) were prepared for analytical ultracentrifugation (AUC) in a buffer containing 50 mM Tris, 50 mM NaCl, 5 mM MgCl_2_, and 1 mM DTT at pH 8, with or without nucleotides (10 μM GTP and 50 μM dATP). AUC and size-exclusion chromatography studies were performed on catalytically inactive D311A variants of SAMHD1, with or without additional cysteine mutations. Four samples were analyzed: D311A, D311A/C341S, D311A/C350S, and D311A/C522S. Sedimentation velocity datasets were acquired in intensity mode at 280 nm for 450 μL samples of 5 μM SAMHD1 at 45,000 rpm and 20°C in a Beckman Optima XLA-1 centrifuge equipped with an eight-hole An50-Ti rotor. All data were analyzed with UltraScan-III (Demeler and Gorbet, 2016; Brookes et al., 2010). Additional experimental details are described in Supplemental Information. Size-exclusion chromatography analysis was performed using a Superdex 200 10/300 GL (GE Healthcare Life Sciences) in 50 mM Tris, 50 mM NaCl, 5 mM MgCl_2_, and 1 mM DTT buffer, with or without nucleotides (10 μM GTP and 50 μM dATP).

### Generation of U937 Cells Stably Expressing SAMHD1 Variants

Retroviral vectors encoding WT or mutant SAMHD1 proteins fused to the N-terminal FLAG peptide tag were created using the LPCX vector (Clontech). Recombinant viruses were produced in 293FT cells by co-transfecting the LPCX plasmids with the pVPack-GP and pVPack-vesicular stomatitis virus G protein (VSV-G) packaging plasmids (Stratagene). The pVPack-VSV-G plasmid encodes the vesicular stomatitis virus G envelope glycoprotein, which allows efficient entry into a range of vertebrate cells. Transduced human monocytic U937 cells were selected in 0.4 μg/mL puromycin (Sigma).

### Restriction Assays

Recombinant retroviruses expressing GFP, pseudotyped with the VSV-G glycoprotein, were prepared as described (Diaz-Griffero et al., 2008). For infections, 6 × 10^4^ cells were seeded in 24-well plates and were treated with a 10 ng/mL concentration of either PMA or DMSO for 16 hr. PMA stock solution was prepared in DMSO at 250 μg/mL. Subsequently, cells were incubated with the indicated retrovirus for 48 hr at 37°C. The percentage of GFP-positive cells was determined by flow cytometry (Becton Dickinson). Viral stocks were titrated by serial dilution on dog Cf2Th cells (ATCC: CRL-1430).

### Cellular dNTP Quantification

Cells were pelleted, resuspended on ice-cold methanol, and dried using a speed vac. The dried samples were resuspended in water and analyzed for dNTP content as described (Kim et al., 2012; Lahouassa et al., 2012).

### In-Cell SAMHD1 Oligomerization Assay

SAMHD1 oligomerization was investigated by co-transfecting 293FT cells (Invitrogen) with plasmids encoding FLAG-tagged and hemagglutinin (HA)-tagged mutant and WT SAMHD1 proteins as previously described (Brandariz-Nuñez et al., 2013). SAMHD1 oligomers were immunopurified using anti-FLAG agarose beads and then analyzed by SDS-PAGE and western blotting using either anti-HA or anti-FLAG antibodies.

### In-Cell SAMHD1 S-Glutathionylation Assay

U937 cells stably expressing SAMHD1-FLAG were treated with 250 μM bio-tinylated glutathione (BIOGEE, Thermo Fisher) for 1 hr. Subsequently, cells were lysed and precleared using protein A agarose. Precleared lysates were incubated with anti-FLAG agarose beads or protein A agarose with the rabbit anti-SAMHD1 antibody when appropriate. Beads containing the immunoprecipitates were washed three times. Subsequently, immune complexes were eluted and analyzed by western blotting using anti-FLAG and anti-biotin antibodies.

### Analysis of T592 Phosphorylation in SAMHD1

T592 phosphorylation levels in the WT and cysteine mutant variants expressed in U937 cells were tested by western blotting using anti-phospho-T592 antibodies as previously described (White et al., 2013b). SAMHD1 expression in cycling U937 cells not treated with PMA is low and is significantly increased after PMA treatment. To analyze T592 phosphorylation status in cycling cells not treated with PMA, SAMHD1 was first preconcentrated by immunoprecipitation using anti-FLAG antibodies (Figure 4C). In PMA-treated U937 cells and in THP-1 cells, detection of T592 phosphorylation by WB was performed on crude cell lysates (Figure 4D). See Supplemental Information for additional details.

### Statistical Methods

When replicate experiments were performed, average values and SDs were calculated using built-in mathematical functions in Prism (GraphPad, La Jolla, CA), Excel (Microsoft, Redmond, WA), and MATLAB (MathWorks, Natick, MA). Centers of plot markers are located at the average values, and error bars denote the average ± SD. Descriptions of what n (number of replicate experiments) represents and other details are provided in the corresponding figure legends. No statistical significance calculations were performed for any of the experiments.

## Supplementary Material

1

## Highlights

- SAMHD1 contains three redox-reactive cysteines: C341, C350, and C522
- C341 and C522 are required for HIV restriction by SAMHD1
- C522S mutation abolishes restriction but preserves the dNTPase activity
- Restriction activity of SAMHD1 may be modulated by redox transformations
