# Supplementary material for: Functionality of Redox-Active Cysteines Is Required for Restriction of Retroviral Replication by SAMHD1
Source: Cell Rep. Author manuscript; Available in PMC 2018 Jul 31. (PMC6067006; doi:10.1016/j.celrep.2018.06.090)
Supplement: 1 [file NIHMS983121-supplement-1.pdf]

Cell Reports, Volume *24*

Supplemental Information

Functionality of Redox-Active Cysteines Is Required for Restriction of Retroviral Replication by SAMHD1

Zhonghua Wang, Akash Bhattacharya, Tommy White, Cindy Buffone, Aine McCabe, Laura A. Nguyen, Caitlin N. Shepard, Sammy Pardo, Baek Kim, Susan T. Weintraub, Borries Demeler, Felipe Diaz-Griffero, and Dmitri N. Ivanov

Figure S1.

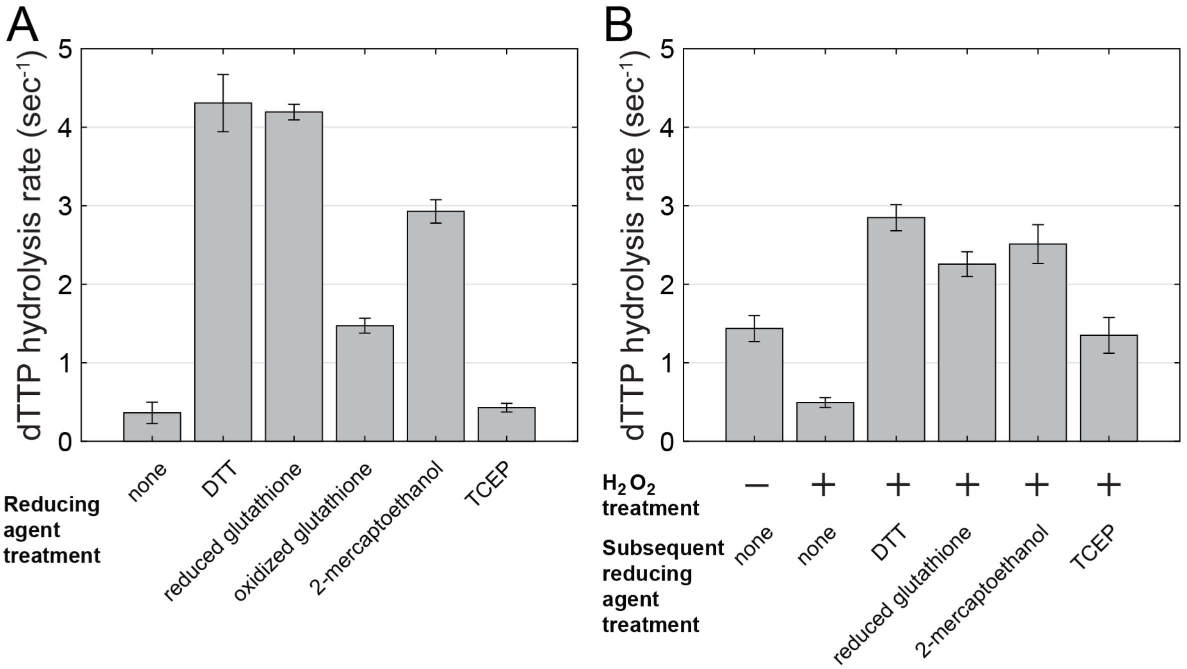

Figure S1. The dNTPase activity of SAMHD1 is affected by the presence of reducing and oxidizing agents. Related to Figure 1. (**A**) Activity of the full-length SAMHD1 purified from mammalian cells is enhanced after 3 min incubation with 5 mM of different reducing agents. (**B**) In contrast, 3 min incubation with 1 mM H_2O_2 causes a reduction in the enzymatic activity that can be reversed with a subsequent treatment with reducing agents. n=2, error bars denote SD.

Figure S2.

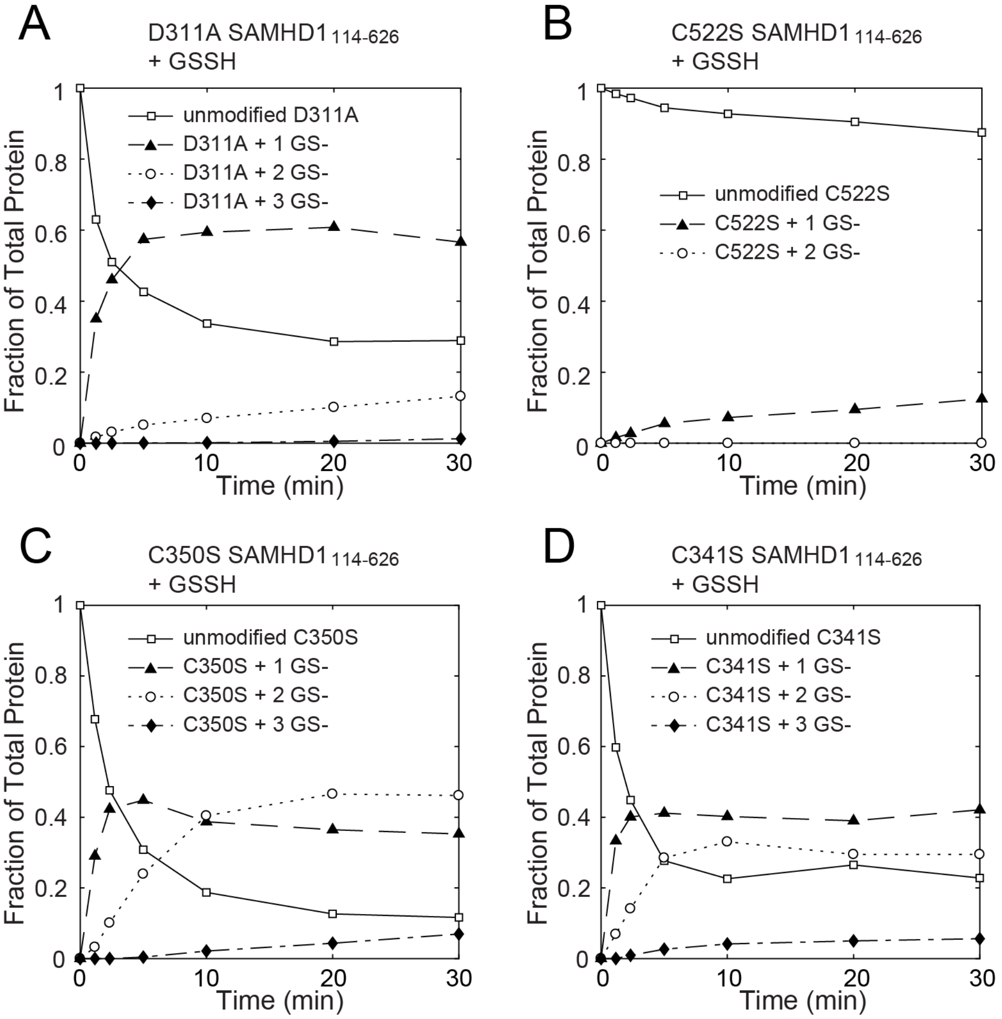

Figure S2. Mutations of redox active cysteins C341, C350 and C522 alter SAMHD1 reactivity with glutathione. Related to Figure 2. Formation of SAMHD1 S-glutathionylated conjugates was monitored by mass spectrometry. (**A**) The active site mutant D311A is rapidly mono-glutathionylated upon incubation with oxidized glutathione (Figure 1E). Di-glutathionylation of the protein occurs at a much lower rate. (**B**) Rapid mono-glutathionylation of SAMHD1 is abolished by the C522S mutation. (**C,D**) The C350S and C341S mutants display an enhanced di-glutathionylation rate. This observation supports existence of the C350-C341 disulfide. When one of the disulfide partners is mutated to serine, the reactivity of the other disulfide partner with glutathione is enhanced and becomes comparable to redox reactivity of C522.

Figure S3.

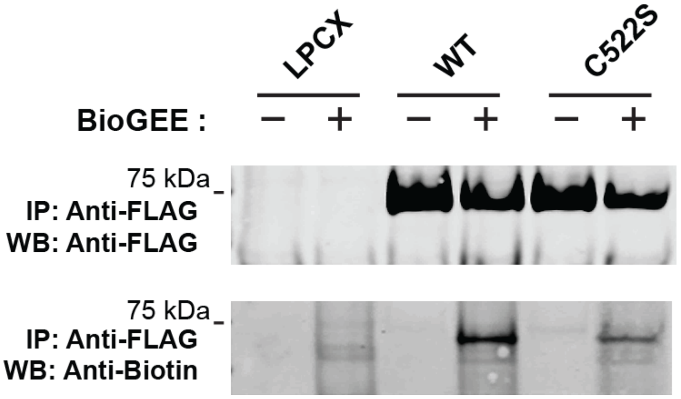

Figure S3. Mutation C522S reduces S-glutahionylation of SAMHD1 in U937 cells. Related to Figure 1 and Figure 2. The effect of the C522S mutation on S-glutathionylation of SAMHD1 in U937 cells was investigated using the same method as in Figure 1G. The observed reduction in C522S glutathionylation is in agreement with the in-vitro glutathionylation experiments shown in Figure S2. Presence of multiple redox-active cysteines in SAMHD1, inherent lability of protein S-glutathionylation and the inability to measure reactivity of specific cysteine residues, limit the utility of WB-based detection of SAMHD1 glutathionylation in cells. In contrast, measurement of SAMHD1 glutathionylation rates and stoichiometries in vitro, as is shown in Figure S2, offer an excellent experimental tool for studies of cysteine redox reactivity.

Figure S4.

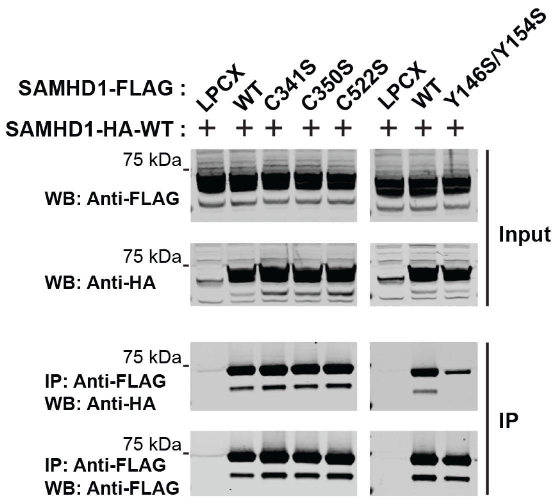

Figure S4. Cysteine mutations do not affect in-cell oligomerization of SAMHD1. Related to Figure 4. Oligomerization of SAMHD1 variants tested by co-immunoprecipitation as previously described (Brandariz-Nunez et al., 2013). Briefly, human 293 T cells were co-transfected with a plasmid expressing wild type SAMHD1-HA and a plasmid expressing wild type or mutant SAMHD1-FLAG proteins. Protein expression was analyzed by Western blotting using anti-HA and anti-FLAG antibodies (Input). Lysates were immunoprecipitated by using anti-FLAG agarose beads and analyzed by Western blotting using anti-HA and anti-FLAG antibodies (IP). As control, we used the SAMHD1 variant Y146S/Y154S, which is defective in its ability to oligomerize (Brandariz-Nunez et al., 2013).

SUPPLEMENTAL EXPERIMENTAL PROCEDURES

Quantification of SAMHD1 S-glutathionylation by intact-mass mass spectrometry

S-glutathionylation of SAMHD1 (Figure 1ABCDEF, Figure 2F and Figure S2) was monitored by intact-mass analysis on an Agilent model 6224 ESI-TOF mass spectrometer used in conjunction with an Agilent 1260 Infinity binary pump HPLC system. The proteins were incubated with 5 mM oxidized glutathione (Acros Organics) at room temperature for variable periods of time as specified in *Results* and then transferred to 1% aqueous acetic acid prior to on-line separation on a C8 column and ESI-TOF/MS analysis. The mass spectra were process by Agilent MassHunter Workstation software (vB.06.00) with deconvolution by the Maximum Entropy algorithm.

Identification of redox-active cysteines by LC-MS/MS

We used a mass spectrometry approach for quantitative analysis of the oxidative state of SAMHD1 cysteines. The strategy relied on the inability of disulfide-linked cysteines to be irreversibly modified by iodoacetamide (Figure 2A). Proteins were first incubated with 5 mM oxidized glutathione for 30 minutes. Samples were then buffer-exchanged using a Zeba desalting spin column (ThermoFisher) into 100 mM ammonium bicarbonate buffer. Protein was denatured by addition of 7 M guanidinium hydrochloride. Protein denaturation was needed to ensure that all reduced cysteines were solvent accessible and modified by iodoacetamide irrespective of their location in the folded protein structure. Free thiols were then alkylated by 1-hr incubation with 10 mM of natural isotopic abundance iodoacetamide ("light"). Only disulfide-linked cysteines remained unmodified during this treatment. Excess iodoacetamide was removed by buffer exchange and disulfide-linked thiols were reduced by 1-hr incubation with 25 mM DTT. Excess DTT was removed by buffer exchange and the protein was treated for 1 hr with 10 mM of "heavy" iodoacetamide (Iodoacetamide-$^{13}C_2$, 2-d_2; Sigma-Aldrich 721328). Samples were then digested overnight with trypsin or chymotrypsin and peptides analyzed by capillary HPLS-ESI-tandem MS on a Thermo Fisher LTQ Orbitrap Velos Pro used in conjuntion with a Sciex/Eksigent NanoLC-Ultra 2-D HPLC system. Data processing was by Mascot (v2.5.1; Matrix Science) and Scaffold (v4.3.2; Proteome Software), using thresholds of 95% peptide and 99.0% proteins in Scaffold. Masses of

cysteine-containing peptides displayed 4 Da difference depending on whether the cysteines were modified during the first or the second iodoacetamide treatment. The ratios of “light” and “heavy” forms of each carbamidomethylated peptide were quantified using Progenesis LCMS (Nonlinear Dynamics/Waters). Based on these abundance ratios all cysteine-containing peptides could be clearly separated into two distinct classes: peptides predominantly modified with the natural-abundance carbamidomethyl group and peptides predominantly modified with the heavy variant (Figure 2D).

Analytical ultracentrifugation

SAMHD1 samples (5 μM) were prepared for analytical ultracentrifugation (AUC) in a buffer containing 50 mM TRIS, 50 mM NaCl, 5 mm $MgCl_2$ and 1 mM DTT at pH 8, with or without nucleotides (10 μM GTP and 50 μM dATP). AUC studies were performed on catalytically inactive D311A mutants of SAMHD1, with or without additional cysteine mutations. A total of four samples were analyzed: D311A, D311A/C341S, D311A/D350S and D311A/C522S. Sedimentation velocity datasets were acquired in intensity mode at 280 nm for 450 μl 5 μM SAMHD1 samples at 45,000 rpm and 20°C in a Beckman Optima XLA-1 centrifuge equipped with an eight-hole An50-Ti rotor. The data were processed and analyzed using Ultrascan III software (Demeler and Gorbet, 2016). The two-dimensional spectrum analysis was used to perform a grid search over user-defined ranges of sedimentation rate and anisotropy to solve a system of Lamm equations which then yields simulated absorbances that are fitted against measured absorbances, simultaneously subtracting time and radially invariant noise from the data (Brookes et al., 2010). Sedimentation velocity results were analyzed by two distinct methods presented in Figure 4CD. Figure 4C shows the model-independent graphical van Holde-Weischet analysis of the data (Demeler and van Holde, 2004, Bhattacharya et al., 2016). Figure 4D shows relative abundance vs. sedimentation coefficient plots obtained by performing a parametrically constrained 2D grid search procedure using the NNLS algorithm in Ultrascan III with 100 Monte Carlo iterations (Gorbet et al., 2014).

Analysis of T592 phosphorylation in SAMHD1

Approximately 1.0 x 10^7 cycling U937 cells (not treated with PMA) stably expressing wild type and mutant SAMHD1 proteins were lysed in 0.5 ml of whole-cell extract (WCE) buffer (50 mM Tris [pH 8.0] 280 mM NaCl, 0.5% IGEPAL, 10% glycerol, 5mM $MgCl_2$, 50 μg/ml ethidium bromide from a 10mg/ml solution [MP; Cat# 04802511], 50 U/ml benzonase [Novagen; Cat# 70746-3], EDTA-free protease inhibitor cocktail tail [Roche; Cat# 11836170001]). Lysates were incubated at 4°C with gentle rocking for 45min and centrifuged at 14,000 rpm for 1 h at 4°C. Post-spin lysates were then pre-cleared using protein A-agarose (bioWORLD; Cat# 20181028-1) for 1 h at 4°C with gentle rocking; a small aliquot of each of these lysates was stored as Input. Pre-cleared lysates containing the tagged proteins were incubated with anti-FLAG-agarose beads (Sigma; Cat# A2220) for 2 h at 4°C with gentle rocking. Anti-FLAG-agarose beads were washed three times in WCE buffer changing the tube between the second and third washes, and immune complexes were eluted using 100μl of 200 μg /ml FLAG tripeptide (Sigma; Cat# F4799) in WCE buffer for 1hr upright with gentle rocking at 4°C. The eluted samples were separated by SDS-PAGE and analyzed by Western blotting using anti-SAMHD1, anti-phospho-T592-SAMHD1 or anti-GAPDH antibodies (Figure 4C).

Analysis of T592 phosphorylation in THP-1 cells and in U937 cells after PMA treatment was performed on cell lysates without prior pre-concentration of SAMHD1 by immunoprecipitation (Figure 4D).

SUPPLEMENTAL REFERENCES

BHATTACHARYA, A., WANG, Z., WHITE, T., BUFFONE, C., NGUYEN, L. A., SHEPARD, C. N., KIM, B., DEMELER, B., DIAZ-GRIFFERO, F. & IVANOV, D. N. 2016. Effects of T592 phosphomimetic mutations on tetramer stability and dNTPase activity of SAMHD1 can not explain the retroviral restriction defect. *Sci Rep,* 6**,** 31353.

BRANDARIZ-NUNEZ, A., VALLE-CASUSO, J. C., WHITE, T. E., NGUYEN, L., BHATTACHARYA, A., WANG, Z., DEMELER, B., AMIE, S., KNOWLTON, C., KIM, B., IVANOV, D. N. & DIAZ-GRIFFERO, F. 2013. Contribution of oligomerization to the anti-HIV-1 properties of SAMHD1. *Retrovirology,* 10**,** 131.

BROOKES, E., CAO, W. & DEMELER, B. 2010. A two-dimensional spectrum analysis for sedimentation velocity experiments of mixtures with heterogeneity in molecular weight and shape. *European Biophysics Journal,* 39**,** 405-414.

DEMELER, B. & GORBET, G. 2016. Analytical Ultracentrifugation Data Analysis with UltraScan-III. Ch. 8, In Analytical Ultracentrifugation: Instrumentation, Software, and Applications. Eds: Uchiyama S., Stafford W. F. and T. Laue. Springer. 119-143.

DEMELER, B. & VAN HOLDE, K. E. 2004. Sedimentation velocity analysis of highly heterogeneous systems. *Anal Biochem,* 335**,** 279-88.

GORBET, G., DEVLIN, T., HERNANDEZ URIBE, B. I., DEMELER, A. K., LINDSEY, Z. L., GANJI, S., BRETON, S., WEISE-CROSS, L., LAFER, E. M., BROOKES, E. H. & DEMELER, B. 2014. A parametrically constrained optimization method for fitting sedimentation velocity experiments. *Biophys J,* 106**,** 1741-50.
